# The Relation of Modern Therapeutics to the Practice of Dentistry

**Published:** 1895-03

**Authors:** Robert W. Greenleaf

**Affiliations:** Boston, Mass.


### THE RELATION OF MODERN THERAPEUTICS TO THE PRACTICE OF DENTISTRY.[1]

BY ROBERT W. GREENLEAF, M.D., BOSTON, MASS.[2]

Mr. Chairman and Members of the American Academy of Dental Science,—For the physician and the dentist, as for the artist, it is well from time to time to stand away from his work, to view its special details from a distance, to ascend a hill-top as it were, and to look forth on the surrounding country. Surely such a view can hardly fail in giving one, who approaches it understandingly, a clearer knowledge of the true perspective and relations with adjacent fields borne by one's special work.

It is in this spirit that I accept your invitation this evening to consider with you from the hill-top of this meeting some of the relations existing between our adjacent fields of thought. What, then, is there in modern therapeutics likely to have special relations to the practice of dentistry. Consider for a moment what modern therapeutics really is. We may do this best by contrasting it with historical therapeutics. In early days medicine passed through a stage which, as Brunton well says, may be called the "make believe" stage of medicine. Just as a child peoples the woods with goblins and various queer creatures, all real to him, and just as he acts towards these according to his belief, so physicians

---

[1] Read before the American Academy of Dental Science, Boston, October 3, 1894.

[2] Professor of Materia Medica, Massachusetts College of Pharmacy.

of olden times persuaded themselves of the reality of their delusions and acted accordingly. Indeed, not a few, calling themselves physicians, are to-day to be classed in this category. I need not delay you with illustrations of this phase of medicine from the past; simply let me give you one quite possible illustration as it may be seen to-day. Two physicians viewing a patient possessing somewhat indefinite symptoms,—we will say cough, pains in the bones, headache, and perhaps a seeming rose-spot or two,—the one hastily concludes that the patient's disease is typhoid fever. He pictures intestinal ulcers and the various train of lesions he has heard result from this invader. He feels sure of this, and treats him accordingly. Another, only partially trained in his profession, equally satisfied with deficient data, equally inclined to hasty conclusions, to "make believe," as it were, is equally *sure* that the patient is suffering with tuberculosis, and treats him accordingly. Both cannot be right; one must be wrong, and perhaps both are.

From the various phases of "make believe" in medicine, as of the savage with his fetiches and charms, through those of the "isms" and "pathies" of to-day, up to those permitting such an advanced illustration as the one I have just given has come instead an attitude towards modern therapeutics as towards all objective questions, permitting the "I know" or "I do not know," "it is" or "it is not" of modern science.

One intermediate phase of an attitude of thought towards objective knowledge is worthy of note,—viz., the "empiric" method. It has given us much of our knowledge of drugs and pointed the way to many of the inquiries of modern therapeutics. The "empiric" is not content with the "make believe" attitude of thought which accepts a statement as true simply because it seems true to the thinker. He values a fact according as it has happened in the same way in repeated instances, even if he does not understand the "why."

It is obvious that empiricism has given us much of value. Nevertheless, such an attitude has retarded the history of medicine, and is not the attitude of to-day. It cannot reach out into the previously unknown. It cannot explain and say why other remedies may not act equally well. While "empiricism" is a step in therapeutic progress far in advance of the "make believe" phases of ancient medicine or of any form of pseudo-medicine existent today, it is inadequate. I can urge upon you no more important truth from a comparative study of the methods for advancing knowledge in our respective lines of thought than this: in all that pertains to your field let your studies be of this third, this modern type of thought and in the spirit of scientific inquiry. Avoid the "make believe" in whatever guise you see it. Put up with the "empiric" only until you can train the scientific search-light upon the special problem. In this way and in this way alone can you solve the problems of your specialty. It is sheer folly and idle waste of time to hold discussions in the "make believe" attitude of thought. Understand me, however; do not undervalue any question of itself. Nothing can be too small or nothing too large, if it come at all within the powers of human thought, for us to give time to its consideration. It is only of the method of its study that I am speaking. For example, in the one case your associate of the "make believe" type of thinkers says grape acids are injurious because so-and-so's teeth decayed after eating grapes. Perhaps the relation is of effect and cause, perhaps not; that I leave for you to tell me. I use this simple illustration solely to contrast the two types of mind. The scientific student will not rest content, because his patient's teeth decayed after eating grapes, that grapes caused the decay. He will inquire how long has she been eating grapes? What is the state of her health in other directions? Can there be any other cause acting or liable to cause decay? How much acid is there in grapes? Where is it, in the skin or in the pulp? What are its properties, chemical, physical, and otherwise? What are its relations to the constituents of teeth outside the body? How may these relations be modified by the presence of saliva? What are its relations to saliva? etc. When he has answered these and perhaps as many more questions from accurate and painstaking study then he can give you an opinion that is worth listening to; moreover, one not likely to result in acrimonious, heated discussion, but likely to bring the interest and peaceful calm inherent to truth and deep conviction.

To take other questions from your specialty. Which of these methods of studying preparatory to discussions would you prefer when discussing such questions as, What are the best materials or operative procedures for bridge-work? Or, What is the best treatment for pulpless teeth? Or, To what extent is it desirable to "treat" a tooth previous to the introduction of permanent fillings? Or, Is there anything in the idea advanced by some dentists that the mercury or other mineral constituent of amalgam fillings can possibly exert a deleterious effect on the system from its presence, or a possibility of an infinitesimally small amount being absorbed? Whether this obtundent is better than that? etc.

I think you will all agree with me that discussions of any such questions, carried on from the "make believe" attitude of thought, of which I have been speaking, are valueless for advancing knowledge; that from the "empiric" attitude they can have but a limited value, and that the road to this end is that of exact observation, neglecting no point, however minute, following out to the last degree the details of accurate, painstaking experiment; in other words, adopting the methods to-day in vogue in all departments of science.

Let me now point out to you some of the facts ascertained in therapeutics by investigations conducted in this spirit, and see if some of them may not be made use of more fully in your practice than is now the case.

Pharmacology has revolutionized our knowledge of drugs. Of the several things it has accomplished these stand out conspicuously,—

1. By careful chemical examination it has isolated the active principles of many drugs, thus permitting their administration without the accompaniment of inert or deleterious principles.

2. By careful study of the principles thus isolated it has been found possible to produce synthetically similar, in some cases more potent or more conveniently obtained, active principles.

3. By careful experimental study of the action of drugs on animals a correct understanding of their action and a degree of exactness of dosage have been obtained, which have placed the therapeutics of to-day to a great extent on a par with other branches of science.

Biology, in both its botanical and zoological relations, has contributed to this end, as well as chemistry. An acquaintance with the life-history of the cells of a complex organism, repeating with modifications the life-history of the cell when forming the entire organism, as with the amœba, has paved the way for knowledge of this character.

The therapeutist of to-day is thus enabled to classify his weapons in the battle against disease with some approach to a scientific system. Moreover, just as he has seen opium displaced from its former high rank as an almost universal panacea for human pains he has learned that drugs are only one form of weapon. Though it is with them alone that I wish to occupy your attention this evening, I wish to make myself clear that the therapeutist regards drugs only at their relative value; that he recognizes the strength of his other weapons, such as the great importance of his surgical appliances and operations, the efficiency of heat and cold as remedial agents, of pure hygienic measures, such as food and rest, of electricity, of massage, and of that latest of acquisitions, hypnotism, which, always at hand, and in one form or other used for centuries by persons in and out of medicine, yet waiting till to-day to be taken out of the bondage of ignorance and viewed calmly in the light of science for what it is worth.

Restricting our view from all these collateral fields of modern practical medicine, let us come now solely to the landmarks of therapeutics with which this discussion is primarily concerned.

Pharmacology enables us to classify medicine into two general groups, which we may call, in the language of botany, divisions. These are medicines acting *systemically* and those acting *extraneously*. The anodynes, the stimulants, the cathartics, etc., are illustrations of Division I. Disinfectants, anthelmintics, etc., are illustrations of Division II. These divisions are again divisible into smaller groups,—viz., the classes *general* and *local*, and each in turn is still further subdivisible into its respective subclasses.

Of the systemic subclasses of a general action we have three very natural representatives,—viz., (*a*) nervines or drugs acting to modify the action of the various parts of the nervous system; (*b*) cardiants or drugs similarly acting on the heart and circulatory organs; (*c*) nutriants or drugs which act conspicuously in influencing the nutrition of the body, as is the case with some of the various tonics, antiperiodics, etc. Each of these subclasses is similarly subdivided into its respective smaller groups, which are not inaptly termed "families" by certain writers,—*e.g.*, the nervines include antispasmodics, anæsthetics, somnifacients, delirifacients, excito-motors, and depresso-motors.

Illustrative of the subclass of locally-acting medicines are some astringent drugs, most cathartics and emetics, also rubefacients, escharotics, etc. A single illustration will serve to indicate how these drugs are studied in order to ascertain their action. Nux vomica, for example, is a drug for a long time known empirically to have poisonous properties.

Chemists isolated its alkaloids, strychnia and brucia, made salts of them, crystallized them, found that they existed in the plant in combination with an acid, igasuric acid, and studied them carefully in all their chemical relations.

Then the physiologist and the pharmacologist took these products and administered them separately and in combination, in large and in small doses, to various animals. It was noticed that most were thrown into convulsions, just as in the case with man; that death would result from a certain large dose; and that at the other extreme—*i.e.*, on the administration of smaller and smaller doses—the effects were less and less marked until a time came when no appreciable effects were produced. It was further noted that when single small doses were administered the effects of the drug ceased after a time, and that this cessation was coincident with its elimination. Then came a series of experiments on how to produce these convulsions. These demonstrated the conclusion that strychnia, besides other actions, produces its effects by direct stimulation of the anterior part of the spinal cord. No one can follow these experiments without feeling the greatness of the human mind which can reach truth through such intricate paths; now the physician, armed with such a knowledge of the action of this drug, applies it to conditions to which the pathologist has given him the key. Thus armed he can use his weapon effectively and safely. He can stimulate the flagging nerves of any organ. It may be the intestine, when the symptoms of disturbed digestion have brought the patient to him. It may be the flagging heart or lungs just ready to give up the battle in the crisis of acute disease. With this powerful stimulant, or with atrophia, digitalis, or morphia, he is sometimes privileged even to stand between a patient's life and death.

We come now to a more special, a still nearer view of drugs: descending our hill-top as it were, to see close at hand which of these groups are especially worthy of the dentist's study. It is obvious that to-night we cannot pick apples from every tree in the orchard of the therapeutic families which would serve your purposes, but let us look at a few of them in the moments that are left us.

The family of antiseptic drugs is certainly worthy of most careful study. The painstaking work of the bacteriologist has shown us how intimately inflammatory processes are connected with the presence of micro-organisms, and he, in connection with the pharmacologist, has given us a long list of drugs which arrest or destroy their activity. Besides corrosive sublimate and other powerful germicides, destructive to our instruments as well as to the germs, they have given us a host of others, as the volatile oils, etc., harmless, even agreeable to our patients, yet serving their purposes admirably. No dentist can turn to the great treatise of Sternberg on bacteria, or to the masterly work of Miller ("Die Microorganismen der Mundhöhle)," or to various other recent works on this subject, without finding much value for use in his daily work.

More than this, we are made familiar with the principles that "asepsis" is what is aimed at, and that soap and water and the most scrupulous cleanliness are essential. Impressed with these results of the pharmacologist's studies, the modern dentist cares for himself, his instruments, and all his equipment, with such a regard for neatness, cleanliness, and the most rigid asepsis and antisepsis that the most painstaking surgeon might well view the appliances with pride.

Let us glance at another tree of our therapeutic garden,—viz., that of anæsthetics. Surely here again we have a family of drugs of especial value to the dentist. Nitrous oxide has done yeoman service for many a year; but would it not be well to give a little more care to the study of ether and chloroform, to know not merely that these powerful anæsthetic agents are dangerous weapons, but also wherein their danger lies?

It seems to me that every dentist should understand the uses and dangers of anæsthetics, also their indications and contra-indications; then, if he chooses, he may use them safely. If he will not learn these matters he must be regarded as criminal to dare to use such weapons. Various new anæsthetics, as bromide and chloride of ethyl, bromoform, etc., are coming into medicine, which may or may not replace our old friends. One other anæsthetic, possessing potent properties of another character,—viz., the local anæsthetic cocaine, which is a delirifacient as well,—deserves more than a passing mention. This extraordinary drug, one of the most useful for performing minor operations, is a dangerous weapon. Though most dentists are deterred from using it at all, I am informed that some are using it with a rashness unheard of in surgery, for the surgeons have learned to fear its immediate toxic action, and find it capable of inducing a "habit" more treacherous, more deadly, than those of alcohol and opium.

This question of "obtundents" is attracting much attention in dentistry at present. Numerous papers are appearing on this subject. It is an auspicious sign. It means that the dentist is rising above the mere mechanical routine of his art, and is seeking what else he may do in the service of his patient.

Closely allied to the family of anæsthetics are the somnifacients and antispasmodics. Of the former we have opium, chloral, and the like. Of the latter, several drugs, as assafœtida, musk, valerian,—relics of the historical days of medicine. Let us include among them also, for the purpose of the point I would present to you, the members of the group of antipyretics, such as antipyrin, acetanilide, and phenacetin, which possess as well anodyne properties. Not to weary you with the details of their special powers, let us consider for a moment the question, How far is a dentist justified in their use? I understand that it is the exception to advise the use of any of these drugs. This appears to be a wise course, inasmuch as prescribing should be undertaken only after an earnest and a careful study of underlying physical conditions, to the consideration of which a dentist can hardly devote sufficient time, even though he be trained for the task. I should place his attitude towards this part of the question on the same basis as that of other specialists, such as aurists and oculists,—viz., the ability to recognize in a general way that a need exists; then, if best, to advise obtaining the proper counsel from the specialist competent to give it.

To obtain such an ability, it is necessary to study the fundamental principles of medicine, as one is not likely to see things of which he has no knowledge.

With it, however, the medically-trained dentist will find many cases in which his patients are needlessly suffering, because they are not given some antispasmodic, tonic, or other aid,—hygienic or otherwise,—indicated by their condition. Moreover, with such knowledge the dentist will find that he is far better equipped for understanding the problems of his own specialty.

Such broader knowledge is to-day considered so desirable that the best dental schools have endeavored to incorporate it among their requirements.

Let us return for a moment to a consideration of still other groups of drugs. Some of them have no obvious relations with dentistry in its more special fields. One may ask why a knowledge of expectorants, sialagogues, and the like, is needed. For reasons already indicated, I consider that such knowledge should form a part of his general training.

However, for mere utilitarian reasons, though such as not of the highest, it may be well to know that sialagogues, such as cubebs and pellitory, might relieve a toothache, particularly of a rheumatic type, simply by locally relieving a congestion. Take mercury also, to which we have already referred,—that is a sialagogue as well as an alterative and antiseptic, and it is well to understand the properties of a drug you are thrown in contact with in so many ways.

We may pass such families as emetics, cathartics, and anthelmintics without especial reference to-night, except to note that each contains some drug, the need of which may be clearly indicated in certain patients under your care, who, but for your friendly counsel, might go unrelieved for years, or suffer serious illness which might have been prevented.

Astringents and rubefacients each have representatives which may serve a dentist well, as in the component parts of tooth-powders, or in special washes for the gums. Illustrations of their usefulness are, doubtless, familiar to you all. The groups of protectives and absorbents also have their respective members, which are useful handmaids of dentistry; but I need not prolong the list.

What I would emphasize to-night I would briefly summarize as follows: In going back to your special field, the lesson may be discussed from the field of modern therapeutics, that more knowledge is to come, not by approaching it as does the "make believer," or the "empiricist," but as the therapeutist is doing,—viz., from the standpoint of the modern scientist, whether he be the chemist, the botanist, or even the psychologist.

Furthermore, I would emphasize that the therapeutist, by study of such a character, has ascertained many facts, has classified them and arranged them for your service; that some of them are already your daily servants; that even of these there are some points as yet unused which you would doubtless find of value; then that there are other agents, a knowledge of which should form a part of your professional training.

These, then, are among the questions which, viewed in the field of therapeutic knowledge, are seen to have important relations with those of the allied field of dentistry.

